# Transforming the health system in Gombe State: accomplishments, challenges and the next frontier

**DOI:** 10.11604/pamj.2026.53.47.50526

**Published:** 2026-01-30

**Authors:** Habu Dahiru, Gafar Bolaji Alawode, Suraj Abdulkarim, Abdulrahman Shuaibu, Abubakar Musa, Ismail Olajide Abdulrazak

**Affiliations:** 1 Gombe State Ministry of Health, Gombe State, Nigeria,; 2 Development Governance International Consult, Federal Capital Territory, Abuja, Nigeria,; 3 Gombe State Primary Health Care Development Agency, Gombe State, Nigeria,; 4 Gombe State Contributory Health Care Management Agency, Gombe State, Nigeria

**Keywords:** Universal health coverage, health sector reforms, primary health care, health financing, human resources for health, sector-wide approach

## Abstract

Nigeria's health reforms aim to accelerate progress toward Universal Health Coverage (UHC). In alignment with these efforts, Gombe State has leveraged national policy frameworks to implement health sector reforms that have improved maternal, newborn, and child health outcomes. To consolidate gains and define future priorities, the State convened the Maiden Gombe State Health Summit in October 2025. The Summit was a two-day hybrid event with over 500 participants from government, development partners, civil society, academia, and the private sector. Data were drawn from rapporteur reports, recordings, presentations, session transcripts, keynote speeches, and policy documents. A thematic analysis approach was used to synthesize key discussions and outcomes. The Summit highlighted strong political commitment, revitalization of 228 primary health centres, upgrades to secondary facilities, expanded immunization coverage, and strengthened disease surveillance. Health workforce reforms, including Human Resources for Health (HRH) governance structures, biometric attendance tracking, training programmes, and welfare improvements, enhanced accountability and productivity. Financial protection was strengthened through the Gombe State Contributory Health Insurance Scheme, covering over 380,000 residents. Implementation of the Sector Wide Approach (SWAp) improved coordination of partner investments. These reforms contributed to measurable reductions in under-five, infant, and child mortality between 2018 and 2023. Gombe State's experience demonstrates how subnational governments can advance UHC through political leadership, alignment with national reforms, primary healthcare strengthening, health workforce investment, sustainable financing, and inclusive stakeholder engagement. The lessons from the Summit provide actionable insights for sustaining reform momentum and strengthening health systems in similar settings.

## Conference proceedings

### Introduction

Nigeria has recorded significant progress in its trajectory towards achieving Universal Health Coverage (UHC) through the creation of several policies and reforms aimed at protecting citizens from financial hardship in accessing healthcare. This includes the National Health Act (2014), which established the Basic Health Care Provision Fund (BHCPF) to expand access to quality health services and strengthen financial risk protection, particularly for poor and vulnerable populations [1,2]. The National Health Insurance Authority (NHIA) Act (2022) further strengthens financial protection by mandating health insurance coverage for all Nigerians [3]. Central to these health reforms is the Nigeria Health Sector Renewal Investment Initiative (NHSRII), which aims to transform the health system and accelerate progress toward UHC by 2030. The Sector-Wide Approach (SWAp) complements these initiatives by reducing fragmentation, improving efficiency, and enhancing service delivery outcomes through pooled financing from government and development partners. Collectively, these policies reinforce Nigeria's commitment to improving access to quality healthcare services with financial protection [1-4].

Gombe State has leveraged these national frameworks to achieve substantial health system gains over the past decade. Between 2018 and 2023, the State recorded notable declines in child, infant, and under-five mortality rates despite enduring challenges such as insecurity, weak infrastructure, and workforce shortages. Specifically, under-five mortality decreased from 189 to 157 deaths per 1,000 live births, infant mortality fell from 104 to 81 per 1,000 live births, and child mortality reduced from 95 to 83 per 1,000 live births [5]. These achievements were catalysed by the 2019 declaration of a State of Emergency in the health sector and the implementation of a multisectoral reform agenda under the Gombe State 10-Year Development Plan.

The State's reforms were aligned with national policies, using BHCPF resources to revitalize 228 primary health centres (PHCs) and upgrade secondary and tertiary facilities, including the construction of a modern 250-bed hospital in Kumo, now designated as a Federal Medical Centre. Workforce strengthening initiatives mirrored national Human Resources for Health (HRH) priorities, incorporating recruitment and deployment of community health workers and midwives, and the introduction of biometric attendance technology to improve accountability and reduce ghost workers. Financial risk protection was expanded through the Gombe State Contributory Health Insurance Agency (GoHealth), covering over 380,000 residents, including vulnerable populations, in line with the NHIA mandate for universal coverage. Strengthened disease surveillance systems and digital health solutions further enhanced health system resilience and data-driven decision-making, reflecting the principles of NHSRII and SWAp. These innovative approaches have earned Gombe State national and international recognition. In 2024, the State received the PHC Leadership Award for Health Innovation and was acknowledged at Chatham House in the United Kingdom for its exemplary health sector reforms [6].

To consolidate these gains, address remaining challenges, and chart the next phase of reforms, the Government of Gombe State convened the Maiden Gombe Health Summit in October 2025. The Summit brought together national and global health leaders, development partners, civil society, academia, and private-sector stakeholders to deliberate on strategies to sustain progress and accelerate UHC. This article presents the proceedings, outcomes, and strategic insights from the Summit, highlighting key priorities for sustaining and scaling Gombe State's health system achievements in alignment with national and global health agendas.

### Methods

**Setting and scope:** the Summit was a two-day hybrid, multistakeholder convening held at the Gombe International Conference Centre in October 2025. It engaged over 500 participants representing high-level dignitaries, government ministries, departments, and agencies (MDAs), development partners, academia, health institutions, civil society organizations, and the private sector. The dignitaries in attendance include: Her Excellency, Senator Oluremi Tinubu, First Lady of the Federal Republic of Nigeria; His Excellency, Alhaji Inuwa Muhammad Yahaya, Executive Governor of Gombe State; Professor Muhammad Ali Pate, Honourable Minister of Health and Social Welfare; Her Excellency, Hajiya Asma'u Inuwa Yahaya, First Lady of Gombe State; Professor Manasseh Daniel Jatau, Deputy Governor of Gombe State and Professor Ibrahim Abubakar Njodi, Gombe State Secretary to the State Government

**Discussion plan:** the Summit was anchored by a well-structured agenda that provided clear direction for all sessions. A panel discussion guide containing targeted, thematic questions was used to engage each panellist, helping to elicit their insights and perspectives throughout the discussion sessions.

**Data sources and collection:** data for this article were sourced from rapporteur documentation, audio recordings, presentation slide decks, session transcripts, goodwill messages and keynote speeches, and relevant background policy documents.

**Analysis:** a thematic analysis approach was employed to synthesise contributions across all sessions. The proceedings were organized into major thematic areas aligned with the Summit's focus on accomplishments, challenges, and future directions.

**Ethical considerations:** all speakers consented to the use of their contributions for documentation and publication. No personal data was collected.

### Results

This section presents the proceedings and outcomes of the Maiden Gombe State Health Summit, synthesizing the deliberations, presentations, and insights shared by speakers, panelists, and participants. The findings are organized into three overarching thematic areas - Accomplishments, Challenges, and the Next Frontier, which together reflect the progress made, the constraints encountered, and the strategic priorities identified for sustaining and accelerating health sector reforms in Gombe State. These themes distil the core messages and lessons from the Summit and provide a coherent basis for guiding future policy decisions, stakeholder engagement, and the continued advancement of UHC in the State.

**Accomplishments:** the Maiden Gombe State Health Summit highlighted substantial achievements across the health system, driven by strong political commitment and alignment with national reforms, including the National Health Act, the BHCPF, and the NHIA mandate for universal insurance coverage [1-3]. Since the declaration of a State of Emergency in the health sector in 2019, the State has implemented comprehensive reforms that strengthened primary healthcare, expanded service coverage, and improved financial protection for its population.

A cornerstone of these accomplishments is the revitalisation of 228 primary healthcare centres (PHCs) across all wards, alongside notable upgrades at secondary and tertiary levels, including the construction of a modern 250-bed hospital in Kumo, later designated as a Federal Medical Centre. These investments contributed to measurable improvements in maternal, newborn, and child health outcomes, consistent with trends reported in the National Demographic and Health Survey (NDHS) 2023-24 [5]. Expanded infrastructure has improved geographical access to essential services and strengthened the continuum of care.

Health financing reforms produced significant gains, particularly through the expansion of the GoHealth, which now covers more than 380,000 residents, including over 100,000 vulnerable individuals, supporting financial risk protection and aligning with the NHIA mandate for universal health coverage [3]. The State also increased its health budget allocation from single digits to 13%, with a target of 15% by 2026, reflecting global calls for strengthened domestic financing to mitigate declining external aid [4,7]. Improvements in public financial management enhanced transparency, accountability, and efficient resource use.

Human Resources for Health (HRH) reforms remain one of the State's most significant achievements. Gombe established comprehensive HRH governance structures, including an HRH Observatory, Steering Committees, Technical Working Groups, and LGA-level HRH units to strengthen coordination and planning [8]. Recruitment and deployment of 440 community health workers and 145 midwives supported by Gavi and BHCPF funding improved service availability at PHC level [1,2]. Digital innovations such as the HRH portal, payroll-linked registries, and biometric attendance tracking technology significantly improved workforce accountability, achieving 98.2% staff verification and eliminating 440 ghost workers, resulting in savings of approximately ₦4.5 billion [9-11].

Workforce development also advanced through the expansion of training capacity, including the establishment of the School of Nursing Sciences, scaling ICT-enhanced training from fewer than 200 to nearly 800 trainees, and strengthening housemanship and continuous professional development programmes. Staff motivation has also been improved through welfare measures such as approval of Consolidated Medical Salary Structure (CONMESS) and Consolidated Health Salary Structure (CONHESS) salary structures, rural posting allowances, timely promotions, and pension clearances.

Another major achievement is the strengthened implementation of the SWAp. Through coordinated development of Annual Operational Plans (AOPs), improved partner alignment, and strengthened public financial management systems, the State reduced duplication and enhanced efficiency, reflecting international evidence on SWAp effectiveness [12,13]. These reforms improved service delivery, reinforced transparency, and enhanced health system stewardship.

Collectively, these accomplishments have contributed to significant declines in child, infant, and under-five mortality between 2018 and 2023, consistent with global evidence showing that sustained investments in PHC, HRH, and financial protection improve survival outcomes [14-16].

**Challenges:** despite these achievements, several challenges continue to constrain Gombe State's health system performance. Human resources for health remain inadequate, particularly in rural and underserved communities. While recruitment efforts have improved staffing levels, maldistribution persists, and retention is hindered by limited incentives and challenging working conditions. Many HRH improvements, including midwife and frontline health worker deployments, still rely heavily on donor support, raising sustainability concerns in the context of declining Development Assistance for Health (DAH) [4].

Demand generation and health-seeking behaviours remain suboptimal. Socioeconomic barriers, cultural norms, and limited awareness hinder uptake of essential health services. These challenges manifest in delayed care seeking, low utilisation of preventive services, and variation in service coverage across Local Government Areas (LGAs). Financing constraints pose another critical challenge. Nigeria is projected to face a $400 million reduction in DAH in 2025, which threatens the sustainability of essential services like immunisation, surveillance, and PHC financing [4]. Although Gombe has increased its domestic budget allocation, resources remain insufficient to expand insurance coverage to all poor and vulnerable groups or fully fund priority programmes. Strengthening strategic purchasing, reducing inefficiency, and enhancing domestic resource mobilization remain urgent priorities [17].

System fragmentation and uneven institutional capacity also persist. While SWAp has improved alignment, some partner interventions still operate vertically, limiting synergy and efficiency. Local government authorities continue to face capacity gaps in planning, budgeting, and supervision, constraining their ability to contribute effectively to PHC financing and management [13].

Infrastructure and service delivery gaps remain evident. Some revitalized PHCs lack adequate equipment, staffing, power supply, and connectivity required to sustain digital tools and quality service delivery. Supply chain challenges contribute to periodic stock-outs of essential medicines and consumables, mirroring broader weaknesses in health systems across low- and middle-income countries [6,18-20].

Despite overall improvements in health outcomes, mortality and morbidity levels remain below national and global benchmarks. Persistent inequities driven by poverty, geographic isolation, and gender norms continue to impede equitable access to care, highlighting the need for more targeted interventions and stronger community engagement.

**The next frontier:** looking ahead, the Summit identified strategic priorities for consolidating gains and accelerating progress toward UHC. Central to this agenda is strengthening PHC delivery and integrating it more effectively with secondary-level care. Improving referral networks for Comprehensive Emergency Maternal and Newborn Care (CEmONC), equipping facilities, and scaling digital solutions for real-time data use are essential to improving service quality and health outcomes.

HRH reforms will remain integral to future progress. Key priorities include expanding rural retention strategies, improving incentives for hard-to-reach areas, sustaining structured recruitment pipelines, and scaling digital HRH systems to enhance data-driven planning and accountability [8-11,21]. Investment in training institutions, scholarships, and specialized programmes will strengthen the health workforce pipeline and address critical skill gaps.

Sustainable health financing remains a critical frontier. The State aims to further expand GoHealth coverage, especially for poor and vulnerable groups, enhance strategic purchasing, engage the private sector and philanthropists, and improve spending efficiency [3,17]. Strengthening fiscal frameworks, blocking leakages, and integrating donor transition into long-term planning will help sustain essential health services. Consistent with global evidence, expanding domestic financing remains the most reliable pathway to achieving long-term UHC [22].

Deepening SWAp implementation is another priority. Strengthening LGA capacity for planning, budgeting, and accountability; aligning all partner interventions with the AOP and the state development plan; and institutionalizing shared monitoring platforms will enhance coordination and reduce fragmentation [12,13,23]. Quality of care improvement will also be central. Routine tracking of quality indicators, strengthening leadership and management capacity at the facility level, and leveraging technology to improve transparency and care outcomes will ensure consistent delivery of essential services. Other emerging priorities include establishing a Health Trust Fund to mobilize additional domestic resources, enhancing the ease of doing business to attract private investment, and forging stronger partnerships with local investors and philanthropists to support system-strengthening efforts.

Finally, Gombe State is positioned to serve as a national learning hub for health sector reform. By documenting its experiences and promoting peer learning on PHC revitalization, HRH reform, SWAp implementation, and financing innovations, the State can contribute significantly to national progress toward UHC. These forward-looking strategies represent the next frontier for building a more resilient, equitable, and efficient health system in Gombe State.

### Discussion

The findings from the Maiden Gombe State Health Summit demonstrate how a subnational government can effectively leverage national reforms, political commitment, and coordinated partnerships to achieve meaningful health system improvements. This experience aligns with global evidence showing that decentralized but well-governed systems can strengthen primary healthcare, expand service coverage, and accelerate progress toward UHC in low- and middle-income countries [6,14]. Gombe State's trajectory illustrates how deliberate investment in PHC, health financing, governance, and human resources can collectively transform service delivery and outcomes.

Strong political will emerged as a principal driver of the reforms. The declaration of a State of Emergency in the health sector catalysed multisectoral action and resource mobilization, similar to reform trajectories observed in Rwanda, Ethiopia, and Liberia, where decisive leadership enabled rapid system strengthening and improved population health outcomes [18-20]. Gombe's revitalization of 228 PHCs, improvements in secondary care infrastructure, and expansion of immunization service points reflect global evidence that targeted PHC investments yield substantial gains in maternal and child health, reduce inequities, and enhance health-seeking behaviour. The documented decline in under-five, infant, and child mortality between 2018 and 2023 reinforces the broader evidence that sustained PHC strengthening is a critical pathway for reducing mortality at scale [15,16].

HRH constituted both a major challenge and an area of strategic reform. The establishment of HRH governance structures such as the HRH Observatory, Steering Committees, and LGA-level HRH units aligns with WHO recommendations for improving workforce planning, accountability, and equitable deployment. Gombe's adoption of digital HRH systems, including biometric attendance tracking and payroll-linked registries, mirrors successful approaches in Kenya, India, and Bangladesh where such tools helped reduce absenteeism, curb payroll fraud, and enhance staff productivity [8-11,21]. The elimination of 440 ghost workers and the resulting fiscal savings support global evidence that biometric verification strengthens efficiency and reduces resource leakage in public health systems. Additionally, Gombe's expanded training capacity and rural retention incentives reflect WHO's global guidance on addressing workforce shortages in underserved areas [11,21].

The Summit also contextualized Gombe's reforms within broader global trends in health financing, particularly the decline in Development Assistance for Health (DAH). The projected $400 million reduction in external aid to Nigeria underscores the urgency of strengthening domestic financing, an area where Gombe has made important progress by increasing health budget allocations and expanding financial risk protection through GoHealth. Expansion of health insurance coverage and increased domestic financing mirror successful experiences from other low- and middle-income countries (LMICs) [22]. The scale-up of GoHealth coverage to over 380,000 beneficiaries aligns with experiences from Rwanda and Ghana, where subnational and community-based insurance mechanisms improved service utilization and reduced out-of-pocket spending [18,23]. These developments reinforce global evidence that domestic financing is the most reliable and sustainable path to achieving UHC in the long term [22].

Efficiency-enhancing reforms such as strategic purchasing, improved supply chain governance, and local production of medical commodities were highlighted as essential next steps. These focus areas align with WHO and World Bank recommendations on reducing wastage, optimizing expenditures, and ensuring value for money in health systems [10,22]. The active involvement of civil society in budget monitoring and accountability processes supports international evidence that social accountability mechanisms improve transparency, equity, and resource allocation [24-26].

The Summit further underscored the critical role of SWAp in strengthening coordination and reducing fragmentation. Evidence from Tanzania, Uganda, and Mozambique shows that SWAp can improve government stewardship and harmonize partner support [12,13]. Gombe's implementation of SWAp through joint Annual Operational Plans, integrated monitoring platforms, and alignment with the NHSRII Compact reflects global best practices in sectoral coordination. Multi-stakeholder engagement involving MDAs, development partners, civil society, the private sector, and academia reinforces literature indicating that inclusive governance enhances accountability, legitimacy, and policy coherence [24,27].

Taken together, Gombe State's experience provides an emerging model for subnational UHC advancement in Nigeria. The alignment of national reforms with State-led innovation, strengthened PHC systems, strategic HRH optimization, expanded financial protection, and coordinated sector-wide governance collectively illustrates a viable pathway for achieving sustainable UHC. Consistent with global evidence [7,22], other Nigerian States could draw valuable lessons from Gombe by institutionalizing SWAp, strengthening HRH governance, scaling PHC revitalization, investing in digital and financial innovations, and increasing domestic financing for health.

## Conclusion

The Maiden Gombe State Health Summit highlighted the significant strides the State has made toward achieving UHC within the broader context of national health reforms. Over the past decade, Gombe State has demonstrated that sustained political commitment, strategic alignment with national policies, and innovative multi-sectoral interventions can yield measurable improvements in health outcomes. Through revitalisation of primary healthcare facilities, expansion of secondary and tertiary services, and the establishment of a contributory health insurance scheme, the State has strengthened service delivery and financial protection, resulting in notable reductions in child, infant, and under-five mortality rates. HRH were central to these achievements, with governance structures, digital HRH systems, and workforce development initiatives enhancing staff accountability, productivity, and equitable distribution. Similarly, proactive domestic financing strategies, including the expansion of GoHealth coverage and the efficient use of pooled resources, positioned Gombe State to navigate declining external aid while ensuring the sustainability of health interventions. The operationalisation of the SWAp further facilitated effective coordination among government agencies, development partners, and civil society, reinforcing transparency, evidence-driven planning, and strategic partnerships. The Summit underscored that Gombe State's experience provides a replicable subnational model for other Nigerian States and similar contexts in low- and middle-income countries. Key lessons include the critical role of political will, the importance of aligning State-level initiatives with national policies, the value of investing in human resources and infrastructure, and the necessity of sustainable health financing and inclusive stakeholder engagement. Collectively, these achievements demonstrate that a deliberate, coordinated, and multi-dimensional approach can accelerate progress toward UHC, strengthen health system resilience, and improve population health outcomes.
